# Epidemiology of Melanoma

**DOI:** 10.3390/medsci9040063

**Published:** 2021-10-20

**Authors:** Kalyan Saginala, Adam Barsouk, John Sukumar Aluru, Prashanth Rawla, Alexander Barsouk

**Affiliations:** 1 Plains Regional Medical Group Internal Medicine, Clovis, NM 88101, USA; drsaginala@gmail.com; 2 Hillman Cancer Center, University of Pittsburgh, Pittsburgh, PA 15232, USA; adambarsouk@comcast.net; 3 Beth Israel Deaconess Medical Center, Harvard Medical School, Boston, MA 02212, USA; Jaluru@bidmc.harvard.edu; 4 Parrish Medical Center, Titusville, FL 32796, USA; 5 Allegheny Health Network, Pittsburgh, PA 15212, USA; alexbarsouk@comcast.net

**Keywords:** melanoma, epidemiology, incidence, mortality, prevention, risk factors

## Abstract

Melanoma accounts for 1.7% of global cancer diagnoses and is the fifth most common cancer in the US. Melanoma incidence is rising in developed, predominantly fair-skinned countries, growing over 320% in the US since 1975. However, US mortality has fallen almost 30% over the past decade with the approval of 10 new targeted or immunotherapy agents since 2011. Mutations in the signaling-protein BRAF, present in half of cases, are targeted with oral BRAF/MEK inhibitor combinations, while checkpoint inhibitors are used to restore immunosurveillance likely inactivated by UV radiation. Although the overall 5-year survival has risen to 93.3% in the US, survival for stage IV disease remains only 29.8%. Melanoma is most common in white, older men, with an average age of diagnosis of 65. Outdoor UV exposure without protection is the main risk factor, although indoor tanning beds, immunosuppression, family history and rare congenital diseases, moles, and obesity contribute to the disease. Primary prevention initiatives in Australia implemented since 1988, such as education on sun-protection, have increased sun-screen usage and curbed melanoma incidence, which peaked in Australia in 2005. In the US, melanoma incidence is not projected to peak until 2022–2026. Fewer than 40% of Americans report practicing adequate protection (sun avoidance from 10 a.m.–4 p.m. and regular application of broad-spectrum sunscreen with an SPF > 30). A 2-4-fold return on investment is predicted for a US sun-protection education initiative. Lesion-directed skin screening programs, especially for those at risk, have also cost-efficiently reduced melanoma mortality.

## 1. Introduction

Melanoma is a malignancy of melanocytes, melanin (pigment) producing cells in the basal layer of the epidermis. Melanocytes are of neural crest origin, and therefore express many signaling molecules and factors that promote migration and metastasis after malignant transformation. Despite representing only 1% of skin cancers, melanoma accounts for over 80% of skin cancer deaths [1].

Melanoma can be divided into many clinical subtypes that differ in presentation, demographics, and molecular profile. Among cutaneous melanoma, superficial spreading melanoma (SSM) is the most common type, especially among fair-skinned individuals, and tends to carry a good prognosis due to a low Breslow thickness, which also depends on the earlier time of diagnosis. Acral lentiginous melanoma, which arises from the glabrous skin of the palms, soles, and nailbeds is more likely to arise in darker-skinned ethnicities. More rarely, and likely independent of sun exposure, melanoma can arise from mucosal or uveal tissue [2]. Uveal melanoma has a particularly poor prognosis, with over 50% of patients developing stage IV disease [3].

The incidence of melanoma has increased in developed, predominantly fair-skinned countries over the past decades [4]. Melanoma is now the fifth leading cancer diagnosis in the US [1]. Our review uses 2020 global statistics (GLOBOCAN) for incidence, mortality, and survival. We also present the latest international initiatives for the prevention of melanoma [5]. Trends in epidemiology, the most common risk factors, and the efficacy of preventative initiatives are reviewed below.

## 2. Epidemiology

### 2.1. Incidence

**Figure 1:**
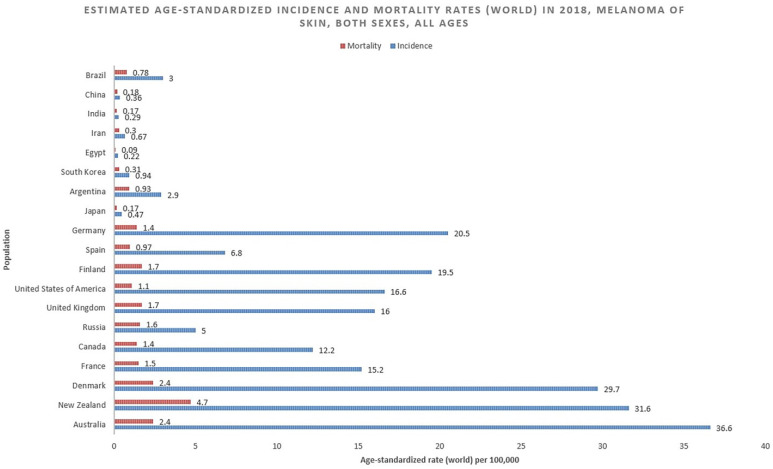
Bar chart showing estimated age-standardized incidence and mortality rates (World) in 2020, melanoma of skin, all sexes, all ages. Data obtained from Globocan 2020 [5].

With an estimated 325,000 new cases in 2020, melanoma of the skin accounts for 1.7% of global cancer diagnoses according to GLOBOCAN (Figure 1) [5]. The age-standardized incidence rate is 3.8/100,000 for males and 3.0/100,000 for females, with cumulative lifetime risks of 0.42% and 0.33%, respectively [4]. 

According to the latest SEER data, melanoma is the fifth most common cancer diagnosis in the US (excluding nonmelanoma skin cancers), with 106,000 estimated new cases in 2021, which represents 5.6% of all cancer diagnoses. Melanoma is particularly prevalent among white males, with an incidence (per 100,000) of 34.7 and 22.1 among white men and women, respectively. For comparison, the male and female incidence was 1.0 and 0.9 among Blacks, and 5.0 for both sexes among Hispanics. The mean age of diagnosis is 65, with 65.7% of diagnoses made in those ages 55 to 84 [1].

**Figure 2:**
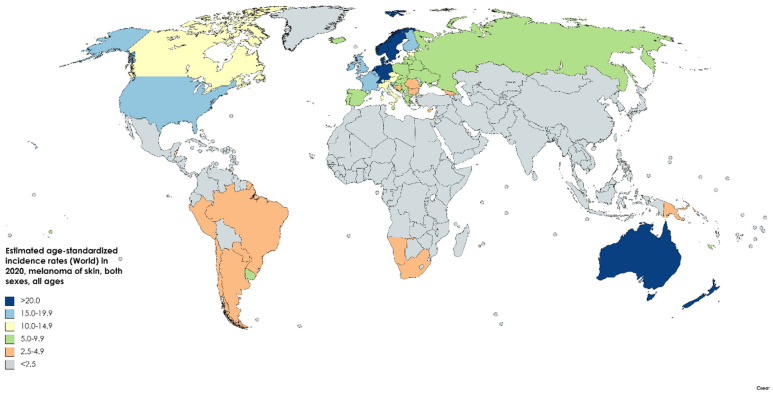
Map showing estimated age-standardized incidence rates (ASR) for melanoma of skin worldwide in 2020, all sexes, including all ages. Created with mapchart.net. Data obtained from Globocan 2020 [5].

Melanoma has seen one of the fastest expansions in incidence among cancers in developed countries (Figure 2). In the US, melanoma incidence grew from 7.9/100,000 in 1975 to 25.3/100,000 in 2018, an over 320% increase. A model in the Journal of Investigative Dermatology recorded the crude incidence as 31.0 among US whites from 2007–2011, projecting an increase to 43.7 by 2027. Meanwhile, the incidence in the UK was found to increase from 5.8 to 19.8 from 1982 to 2011, while Sweden increased from 13.0 to 28.3 and Australia from 26.4 to 51.6. Notably, incidence peaked in Australia around 2005 and is projected to continue declining thanks to effective public health campaigns and increased sunscreen accessibility. While other developed nations are also seeing melanoma growth slowing down, the incidence in the US is projected to peak around 2022–2026, and incidence in Sweden and northern Europe is unlikely to stabilize before 2030 [6].

### 2.2. Mortality

**Figure 3:**
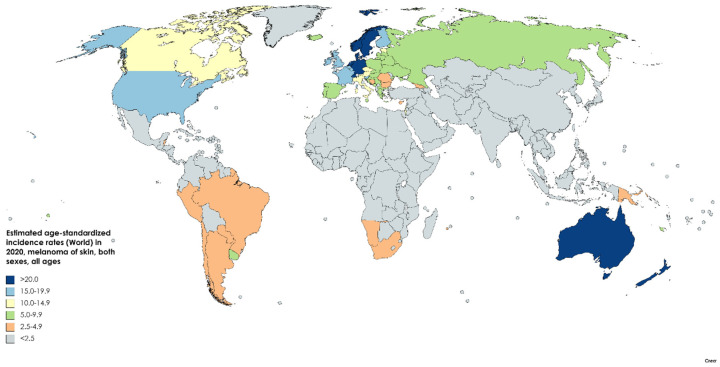
Map showing estimated age-standardized mortality rates (ASR) for melanoma of skin worldwide in 2020, all sexes, including all ages. Created with mapchart.net. Data obtained from Globocan 2020 [5].

An estimated 57,000 people died of melanoma in 2020, according to GLOBOCAN (Figure 3), resulting in age-standardized mortality of 0.7/100,000 for men and 0.4/100,000 for women worldwide [4]. The mortality rate in the US was 2.0/100,000 in 2018, as compared to a high of 2.8/100,000 in 2009. The mortality among white men and women is 3.9 and 1.7/100,000 respectively (vs. 0.4 and 0.3 for Black men and women). Melanoma will account for an estimated 7180 deaths in the US in 2021, with a median age of death of 71, and 66% of those dying older than 65. Melanoma accounts for over 80% of skin cancer deaths [1].

Among whites in the US, mortality from melanoma increased by 7.5% from 1986 to 2013 [1]. However, with the approval of 10 new targeted and immunotherapy treatments since 2011, overall mortality decreased by 17.9% from 2013 to 2016 [7].

### 2.3. Survival

**Figure 4:**
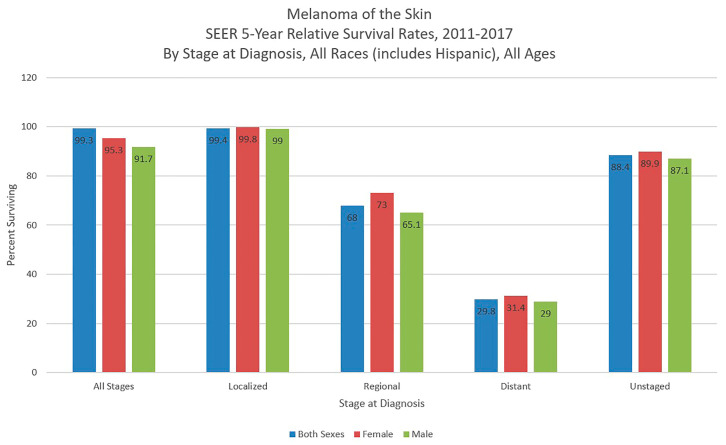
Melanoma of the skin 5-year SEER relative survival rates, 2011–2017 by stage at diagnosis and sex. Data source: US Mortality Files, National Center for Health Statistics, CDC [1].

The most recent 5-year survival rate (2011–2017) according to SEER is 93.3% for melanoma, up from 81.9% in 1975, the earliest recorded. The 5-year survival is 99.4% for those first diagnosed with stage I–II disease, decreasing to 68.0% for stage III and 29.8% for stage IV. Only 4% of diagnoses are made in stage IV, while 83% of diagnoses are stages I-II (Figure 4) [1].

## 3. Risk Factors

### 3.1. Sun Exposure

UV exposure is the primary risk factor for melanoma of the skin, though this effect is heavily modulated by genetics, melanin, and UV wavelengths.

UV light is known to induce DNA photoproducts, most commonly thymidine-dimers, which, if unrepaired by nucleotide excision repair (NER), cause errors in DNA replication, subsequent mutations in cell signaling molecules, and ultimately carcinogenesis [8]. Patients with xeroderma pigmentosum, a hereditary defect in NER, have up to a 20,000-fold increased risk of skin cancer, as well as neuro-degeneration [9,10].

UVB light (wavelength: 280–320 nm) is considered 1000 times more genotoxic per photon than UVA (320–400 nm), although UVA environmental exposure is up to 20–40-times higher depending on time, season, latitude, and altitude [11,12]. UVA exposure is also greater through glass windows or in sunbeds, and most non-broad-spectrum sunscreens do not filter UVA as well as UVB [12]. Unlike UVB, UVA has also been shown to induce oxidative (aerobic) damage to DNA, which is repaired through a different system (base excision repair) than UV-induced pyrimidine dimers [13,14,15]. UVA seems to have a lower rate of DNA repair and a consequently higher rate of mutations per photoproduct in melanocytes [14].

The location of UV-induced mutations, i.e., cancer’s molecular profile, varies highly with melanoma subtype, prognosis, and response to treatment. Commonly mutated proteins include members of the mitogen-activated protein kinase (MAP-K), such as NRAS and BRAF, which are responsible for cell growth and differentiation. BRAF is more commonly mutated in younger patients with more nevi and sun exposure and those with superficial-spreading melanoma (SSM) (up to 50%) and is now targeted with BRAF + MEK small molecular inhibitors as a front-line treatment [16,17,18]. Meanwhile, mutations in the oncogene KIT are seen in 0% of SSM cases but up to 20% of acral lentiginous (ALM) and mucosal melanoma (MM) cases [19]. Uveal melanoma is not associated with chronic sun damage or MAP-K mutations, but rather with mutations in GNAQ and GNA-11 [20]. UV-induced mutations in tumor-suppressor p53 are more commonly seen in those with stage IV disease and are associated with a worse prognosis [15]. NER can also be impaired by UV-induced mutations, increasing the risk of tumorigenesis [8].

Eumelanin, synthesized in greater proportions in dark-skinned individuals, is more protective against UV radiation than phaeomelanin, which is found in greater proportions in those with fair skin and red hair. While eumelanin’s scattering of UV rays protects against DNA damage, it also decreases cutaneous vitamin D3 production, which explains why homo sapiens who left Africa for higher latitudes (with less UV exposure) were selected for phaeomelanin and lighter skin [11,21,22].

### 3.2. Indoor Tanning

An estimated 7.8 million women and 1.9 million men use tanning beds annually, although the International Agency for Research on Cancer (IACR) identified tanning bed radiation as a carcinogen due to higher levels of UVA and UVB exposure than that of the daily sun (for most latitudes) [23]. A dose–response relationship has been noted between total hours spent in a tanning bed, the number of sessions, or years of tanning bed usage, and melanoma risk [24]. Indoor tanning rates among US high school students declined from 15.6% in 2009 to 7.3% in 2015, although white, non-Hispanic young women continue to have the highest rates of usage [25]. A JAMA Dermatology economic analysis from 2020 concluded that banning indoor tanning among those under 35 could avert 448,000 cases of melanomas [26].

### 3.3. Immunosuppression

Low doses of UVA and UVB have also been shown to decrease immunosurveillance by Langerhans and dendritic cells, impairing antigen-presentation and T-cell and NK-cell activation against aberrant melanoma cells. Unsurprisingly, immunosuppressed patients (e.g., iatrogenically or due to HIV) have an increased risk of melanoma [27,28,29,30]. These findings may also explain why melanoma is particularly responsive to checkpoint-inhibitor immunotherapy, monoclonal antibodies that stimulate T-cells to recognize and destroy cancer cells. Ipilimumab (tradename Yervoy), a CTLA-4 inhibitor, received its first FDA approval in 2011, specifically for melanoma, and has since been approved for many other tumor types in combination. PD-1 and PDL-1 inhibitors, such as pembrolizumab and nivolumab (tradenames Keytruda and Opdivo), have since been approved in various combinations for stage III and IV disease. A combination of PDL-1 inhibitor atezolizumab (tradename Tecentriq) with BRAF inhibitor vemurafenib (tradename Zelboraf) and MEK inhibitor cobimetinib (tradename Cotellic) was approved in 2020 for unresectable or metastatic BRAF V600 positive melanoma [31]. In limited, small clinical trials, immunotherapies have been particularly beneficial for uveal melanoma patients, who otherwise have a poor prognosis [32].

### 3.4. Moles (Nevi)

Moles, or nevi, are benign growths of melanocytes considered both direct precursors and markers of increased risk for melanoma. In a population-based study in the US, the annual transformation rate of any single mole into melanoma was found to range from 0.0005% in those under 40 to 0.003% for men over 60. One study found those with >100 moles are at a seven-fold increased risk of developing melanoma relative to those with <15 [33]. The authors concluded that moles that persist into old age are particularly at risk for malignancy [34]. Guidelines suggest these moles should be surveilled based on the ABCDE criteria (asymmetry, border irregularity, color variation, diameter >6 mm, and evolution), and if suspected, resected with margins of at least 2 mm [35].

### 3.5. Family History

Around 10% of patients with melanoma have a family history of the disease, though only a few congenital syndromes, such as congenital nevi and mutations, have been characterized. Mutations in the CDKN2A gene are rare in sporadic cases but have been implicated in up to 30% of hereditary melanomas [36].

Dysplastic nevus syndrome (DNS) is a rare congenital disease of atypical nevi associated with an increased risk of melanoma of the skin, as well as other rare locations, such as melanoma of the gallbladder [37,38,39]. DNS is associated with neurofibromatosis type 1 and several other endocrine disorders [40]. A recent case report also suggests DNS increases the risk of pregnancy-associated melanoma [41]. DNS does not seem to increase the risk of other cancers. Patients with DNS or other forms of familial melanoma must undergo regular screenings of skin and moles and avoid high-risk activities such as UV exposure without sunscreen in order to decrease their risk [39]. Pediatric patients with congenital melanocytic nevi (CMN) are born with or develop many large moles which put them at greater risk of neurocutaneous melanocytosis and melanoma. The most commonly implicated mutations are in NRAS. Constant monitoring and mole resection by an interdisciplinary team is recommended, and trials are currently evaluating prophylactic MEK inhibitors for CMN patients [42].

### 3.6. Obesity

Melanoma incidence may be associated with obesity, with some, but not all, studies showing an increased risk among those with a BMI over 30. However, several recent studies also show improved survival outcomes for obese melanoma patients on targeted treatments and immunotherapies. Excess body fat seems to induce BRAF V600E oncogene activity through metabolic signaling and suppress immunosurveillance, which may explain why obese patients show above-average PFS and OS on BRAF inhibitor therapies and immunotherapies that specifically target these pathways [43,44].

## 4. Prevention

Public health initiatives in some developed nations, such as Australia, have been effective in curbing the growth in melanoma incidence and should be used as models for education and funding in the US.

### 4.1. Primary Prevention and Education

Along with tobacco, obesity, diet, alcohol, and certain viruses, sun exposure contributes to the estimated 45% of cancer deaths that are preventable according to the ACS [45]. Multiple randomized controlled trials have found that regular sunscreen use significantly reduced melanoma rates decades later [46]. The American Cancer Society (ACS) suggests sun avoidance between 10 am and 4 pm, or if not feasible, the usage of hats, clothing, and broad-spectrum sunscreen with a sun protection factor (SPF) of 30 or higher [47]. The ACS also recommends total avoidance of artificial UV exposure, such as tanning beds. However, in a 2018 online panel of over 3000 Americans, only 38.8% confirmed using sunscreen on the face, neck, and chest when outside in the sun, with only 19.9% applying sunscreen to their whole exposed body [23].

The Australian state of Victoria has been running the SunSmart program since 1988, which used television advertising to stress the use of hats and sunscreen. A meta-analysis of nine cross-sectional studies found that sunburn incidence was halved by 2002, and those exposed to the advertising condoned a greater usage of sun protection [48]. The SunSmart school accreditation program required hat-wearing, shade seeking, and positive sun-protective behavior role modeling for grade-school students. The number of Victorian schools with sun protection policies has increased from 17% in 1993 to 89% in 2013 [49].

Although narrower in adoption, the US has also seen success with prevention initiatives. An estimate from 2008 found the SunWise program prevented more than 11,000 cases and 50 deaths from skin cancer, saving an estimated $2–4 for every dollar invested. These returns suggest greater investment in sun-exposure prevention is necessary [50].

### 4.2. Screening

In 2016, the US Preventive Services Task Force concluded that there was insufficient evidence for clinical skin cancer screening for asymptomatic adults without a history of malignancy or skin lesions. However, adults with a family history, genetic predisposition, pertinent past medical history, or history of sun exposure and fair skin are recommended to be regularly screened [51]. A meta-analysis of 15 studies from 2017 found a clinical benefit to skin cancer screening programs [52]. A Belgian study found that lesion-directed skin exams had similar rates of detection to whole-body skin exams, which take six times longer [53]. The evidence for skin self-exam was highly variable, and performance was highly associated with spouse involvement and the availability of a wall mirror [54].

## 5. Conclusions

Melanoma is a leading cancer diagnosis in the developed world and is projected to continue to increase in incidence over the coming decades. Mortality rates have fallen thanks to advances in targeted and immunotherapies, though those diagnosed with stage IV disease still have a dismal survival rate. Prevention remains essential for reducing healthcare costs and minimizing morbidity and mortality. Risk factors such as UV exposure without broad-spectrum sunscreen or other protection, indoor tanning, immunosuppression, and obesity are primary targets for educational programs, which have been highly effective in decreasing melanoma incidence in Australia. Screening is recommended for those with risk factors such as family or prior history, congenital diseases, predisposing lifestyle/occupation, and high-risk demographics, in particular older, white men.

## Data Availability

The data that support the findings of this study are available at https://gco.iarc.fr/today/, reference number [5].
